# Maternal vegetable intake during and after pregnancy

**DOI:** 10.1186/s12884-019-2353-0

**Published:** 2019-07-26

**Authors:** Alison Tovar, Jill L. Kaar, Karen McCurdy, Alison E. Field, Dana Dabelea, Maya Vadiveloo

**Affiliations:** 1 Department of Nutrition and Food Sciences, University of Rhode Island, 41 Lower College Road, Kingston, RI 02881 USA; 2 Department of Pediatrics, School of Medicine, University of Colorado Anschutz Medical Campus, Aurora, CO USA; 3 Department of Human Development and Family Studies, University of Rhode Island, Kingston, RI USA; 4 Department of Epidemiology, Brown University, Providence, RI USA; 5 Department of Epidemiology, Colorado School of Public Health, University of Colorado Anschutz Medical Campus, Aurora, CO USA

**Keywords:** Vegetable intake, Pregnancy, Employment, Maternity leave

## Abstract

**Background:**

Improved understanding of vegetable intake changes between pregnancy and postpartum may inform future intervention targets to establish healthy home food environments. Therefore, the goal of this study was to explore the changes in vegetable intake between pregnancy and the postnatal period and explore maternal and sociodemographic factors that are associated with these changes.

**Methods:**

We examined sociodemographic, dietary, and health characteristics of healthy mothers 18-43y from the prospective Infant Feeding Practices II cohort (*n* = 847) (2005–2012). Mothers completed a modified version of the diet history questionnaire, a food-frequency measure, developed by the National Cancer Institute. We created four categories of mothers, those that were: meeting vegetable recommendations post- but not prenatally (*n* = 121; *improved* intake), not meeting vegetable recommendations during pregnancy and postnatally (*n* = 370; *stable inadequate*), meeting recommendations pre- but not postnatally (*n* = 123; *reduced* intake), and meeting recommendations at both time points (*n* = 233; *stable adequate*). To make our results more relevant to public health recommendations, we were interested in comparing the *improved* vegetable intake group vs. *stable inadequate* vegetable intake group, as well as those that *reduced* their vegetable intake compared to the *stable adequate* vegetable intake group. Separate multivariable-adjusted logistic regression were used to examine sociodemographic predictors of *improved* vs. *stable inadequate* and *reduced* vs. *stable adequate* vegetable intake.

**Results:**

Women with *improved* vegetable intake vs. *stable inadequate* smoked fewer cigarettes while women with *reduced* vegetable intake vs. *stable adequate* were more likely to experience less pregnancy weight gain. In adjusted models, employed women had greater odds of *reduced* vegetable intake (OR = 1.64 95% CI 1.14–2.36). In exploratory analyses, employment was associated with greater odds of *reduced* vegetable intake among low-income (OR = 1.79; 95% CI 1.03–3.1), but not higher income women (OR = 1.31; 95% CI 0.94–1.84). After further adjustment for paid maternity leave, employment was no longer associated with vegetable intake among lower income women (OR: 1.53; 95% CI: 0.76–3.05).

**Conclusions:**

More women with *reduced* vs. *stable adequate* vegetable intake were lower income and worked full time. Improved access to paid maternity leave may help reduce disparities in vegetable quality between lower and higher income women.

## Background

A mother’s diet is important both during and after pregnancy. During pregnancy, maternal diet can influence flavor and food preferences for their infant (e.g., expression of genes related to flavor preferences and greater exposure to flavors through the amniotic fluid and breastmilk) [1]. Maternal diet is also important for the offspring where the shared food environment (i.e., food availability and role modeling) can influence a child’s risk for obesity [2, 3]. Although mother’s overall diet is of importance, vegetables are particularly critical. Vegetable intake during the first year of life is associated with the development of healthy eating habits later in life. Unfortunately, most adults and children fall short of the recommendations. Once complementary feeding has been initiated, 30–40% of children 6 months and older do not eat a vegetable [4, 5]. Early exposure to vegetables may increase the amount and variety of these foods consumed later in childhood [2, 6–10]. Several studies have also found that vegetable intake is a proxy for having a higher diet quality [11–14]. Thus, it is of public health importance to identify changes in vegetable intake between pregnancy and the postnatal period and whether sociodemographic factors predict any of these changes. A better understanding of this possible dietary transition can help identify future intervention targets. In addition, understanding what factors contribute to the transition of improving their vegetable intake or reducing their vegetable intake from pregnancy to postpartum, can better help tailor obesity prevention efforts to pregnant and postpartum women and their children.

Although some studies have explored the transition of a woman’s diet from before to during pregnancy, few studies have looked at how their vegetable intake may change after birth. Research suggests that women adopt healthier eating patterns including eating more fruits and vegetables and less fast food during pregnancy [15]. Most often, women who make these changes are older and more educated [16–18], and these healthy changes are not necessarily sustained after delivery [19]. The few studies that have explored what happens to women’s diets after pregnancy have focused on describing how macronutrients change from pregnancy to 4 and 5 years postpartum [20, 21]. Exploring changes specifically related to vegetable intake, within a shorter time period after delivery may be important for identifying early intervention targets to improve maternal diet, child obesity risk, and the shared food environment early in life. Additionally, exploring changes in vegetable intake rather than macronutrient intake is informative for developing public health recommendations for pregnant and postpartum women. Therefore, the goal of this study was to 1) explore the changes in vegetable intake between pregnancy and the postnatal period in a large, prospective cohort and 2) explore maternal and sociodemographic factors that are associated with these changes. To make our results more relevant to public health recommendations, we described these changes for mother’s; that met vegetable recommendations postpartum but not during pregnancy (*improved* vegetable intake group) as compared to those did not meet vegetable recommendations during pregnancy or postpartum (*stable inadequate* vegetable intake group) as well as those that that reduced their vegetable intake (*reduced* their vegetable intake) compared to those that met vegetable recommendations both during pregnancy and postpartum (*stable adequate* vegetable intake group).

## Methods

We analyzed (2017) secondary data from the Infant Feeding Practice II (IFSP II) study which is a prospective, longitudinal cohort (2005–2012) conducted by the Food and Drug Administration (FDA) and the Centers for Disease Control and Prevention (CDC) in the United States, that followed about 2000 mother-infant pairs from the third trimester of pregnancy throughout the first year of life and then again at 6 years to study a variety of infant feeding practices [22].} Women (*n* = 4900) were drawn from a national consumer panel during the third trimester of pregnancy, with all data excluding the birth data collected by mail questionnaires. Participating women and their infants could not have a medical condition at birth that would affect feeding and the infant had to have been born after at least 35 weeks’ gestation, weigh at least 5 lbs., be a singleton, and not have stayed in intensive care for 3 or more days. After the birth screener, infant-mother pairs were disqualified if the infant was reported to have a serious, long-term health problem that would affect feeding.

A subset of mothers was invited to complete a modified diet history questionnaire (DHQ) prenatally (*n* = 1444, response rate 82.2%) and 4-months postpartum (*n* = 1422, response rate = 79.4%). This 149-item questionnaire is a food-frequency measure developed by the National Cancer Institute [22, 23]. Modifications to this questionnaire included changing the time frame of the DHQ from 1 year to 1 month and adding specific foods of interest for pregnant women, including specific types of fish and specific dietary supplements [24].

Per the IFPS II protocol [22], plausible energy intake in the prenatal sample included women with intakes between 671 and 6265 kcal and 606–4539 in the postpartum sample. We analyzed data from the 847 women who completed the DHQ at both time points. Intake (servings/day) of total fruits, vegetables, dairy, sugar-sweetened beverages (SSB), and added sugars (teaspoons) were calculated from the DHQ. Information about infant feeding practices was self-reported by mothers 9 times over the infant’s first year of life. Additional details about the IFPS II and the prenatal and maternal DHQ subsamples have been previously published [22].

High vegetable intake, defined as meeting the vegetable intake recommendations according to the 2015 Dietary Guidelines for Americans, is both an essential component of dietary quality, favorably associated with pregnancy outcomes, and inadequately consumed among many pregnant [25]. Mothers were categorized into one of four groups: 1) met vegetable recommendations postpartum but not during pregnancy (*n* = 121) (*improved* vegetabl*e* intake group) 2) did not meet vegetable recommendations during pregnancy or postpartum (*n* = 370) (*stable inadequate* vegetable intake group*)*; 3) met vegetable recommendations during pregnancy but not during the postpartum period (*n* = 123) (*reduced* vegetable intake group) and 4) met vegetable recommendations both during pregnancy and postpartum (*n* = 233) (*stable adequate* vegetable intake group); and meeting vegetable intake recommendations was defined as ≥2.5 vegetable servings/day after adjustment for total energy intake using the residual method [26] in accordance with the Dietary Guidelines for Americans [25].

### Sociodemographic and maternal variables

We selected available sociodemographic variables that have been associated with maternal dietary intake or quality pre- and post-partum and which were available in IFPS II [27–29]. All questionnaires were developed by the US Food and Drug Administration in collaboration with the Center for Disease Control and members of the working group who had specific expertise in each topic. The prenatal questionnaire was used to collect information about the women’s health and health care, and employment status using standard questions related to demographics.

### Sociodemographic variables

Self-reported age was assessed during pregnancy. Maternal participation in the Women Infants and Children (WIC) program and employment status (9 options ranging from unemployed, student, homemaker, part-time, and full-time employment) were measured during pregnancy and in the first year of life. Maternal employment was re-categorized as a 3-level ordinal variable capturing unemployed (fulltime homemaker, disabled student, etc. and not employed, retired and not employed, temporarily unemployed), part-time employment (self-employed, works for someone else part time only), and full-time employment. Family poverty income ratio (PIR) was classified as low (< 185% of poverty guidelines) or high (≥185% of poverty guidelines). Maternity leave was computed as the number of weeks of paid leave. After examining missing data in the paid maternity leave variable (50.2% missing), we replaced missing values with a ‘0’ for women who reported anything other than full time employment and had missing data for paid maternity leave since these women would not be eligible for paid leave.

### Maternal variables

Pre-pregnancy BMI was computed from self-reported height and weight just before pregnancy. Participants also reported the current average daily number of cigarettes smoked during pregnancy, whether or not they experienced gestational diabetes with pregnancy (y/n). Total breastfeeding duration was reported by the mother during the month that she stopped breastfeeding entirely and women also completed the Edinburgh Postpartum Depression Scale, a 10-item validated, broadly utilized tool developed to identify women who have postpartum depression [30].

### Statistical analysis

We examined descriptive characteristics among the four groups of mothers and made comparisons according to our objectives of this study using ANOVA, t-tests and chi-square tests for continuous and categorical variables. We first computed an overall ANOVA to assess differences across the four groups and then did comparisons of the groups according to our established research questions: *improved* vegetable intake vs. *stable inadequate* vegetable intake and *reduced* vegetable intake vs. *stable* adequate intake. Given our interest in comparing the groups according to those that improved and those that reduced, logistic regression was used. We examined the associations of sociodemographic factors with *improved* vegetable intake (vs. maintaining a *stable inadequate* vegetable intake) and *reduced* vegetable intake (vs. maintaining a *stable adequate* vegetable intake) adjusted for covariates. Given the wealth of literature exploring socio-economic disparities, in exploratory analyses, we assessed differences in significant predictor variables by income status. To do this, we conducted secondary analyses stratified by poverty income ratio (i.e., higher socioeconomic status (poverty income ratio > 1.85) versus lower socioeconomic status (poverty income ratio < 1.85). We chose this cutoff given the federal nutrition program guidelines such as the WIC program to determine eligibility. We also compared whether other maternal dietary components (i.e., fruit, added sugar, discretionary fat, and dairy) differed between these groups of women to explore how changes in vegetable intake correlated with maternal intake of other dietary components using logistic regression models. Predictors were selected based on their relevance in the previous literature [27, 31, 32], and age and any variable that changed the odds ratio by 10% or more was included in the final model [33, 34]. All analyses were conducted with SAS v. 9.4.

## Results

Close to one-third of women of women (28.8%, *n* = 244) experienced shifts in vegetable intake between pregnancy and postpartum. Less than one quarter of the women did not meet recommendations during pregnancy and subsequently met recommendations during the postpartum period (i.e., *improved* vegetable intake) (14.2%; *n* = 121). Similarly, nearly the same number of women either met recommendations during pregnancy, but not during the postpartum period (i.e., *reduced* vegetable intake) (14.5%; *n* = 123). Overall, more than 40% of the women did not meet vegetable recommendations during and after pregnancy (43.6%; *n* = 370; *stable inadequate*) and less than 30% met recommendations at both time points (27.5%; *n* = 233; *stable adequate*).

Sociodemographic characteristics did not differ among women who *improved* their vegetable intake and those in the *stable inadequate* group. There were significant sociodemographic differences among women who reduced their vegetable intake. Compared to women in the *stable adequate* group, women who *reduced* their vegetable intake postpartum were lower income (PIR = 2.7 ± 1.7 vs 3.1 ± 2.4; p = 0.04) and were more likely to work full time (43.5% vs. 30.0%; p = 0.047, respectively) (Table 1). Women who *improved* their vegetable intake had significantly greater mean fruit (2.5 ± 0.17 vs.1.8 ± 0.2; *p* = 0.0006) and vegetable (2.8 ± 0.10 vs. 1.3 ± 0.06; *p* < 0.0001) intake and reduced sugar intake (49.8 ± 0.23 vs.50.8 ± 0.13 g; *p* < 0.0001) at both time points compared to women in the *stable inadequate* group. Those that had *reduced* vegetable intake, had lower servings of fruit (1.7 ± 0.17 vs. 2.3 ± 0.12; *p* = 0.002), vegetables (2.7 ± 0.10 vs. 4.25 ± 0.07; *p* < 0.001) and whole grains 2.9 ± 0.05 vs. 3.1 ± 0.04; *p* = 0.01 and higher grams of sugar (50.0 ± 0.23 vs. 48.8 ± 0.17;*p* < 0.0001) compared to those in the *stable adequate* group.

**Table 1:** Maternal Sociodemographic Characteristics by Energy-Adjusted Change in Vegetable Intake in the IFPS II

Mean (SD) ^c^ Residual adjusted + 2.5						
	Improved Vegetable Intake (*n* = 121)	Stable Inadequate (*n* = 370)	*p*-value	Reduced Vegetable Intake (*n* = 123)	Stable Adequate (*n* = 233)	*p*-value
Age (yrs.)	28.7 (5.25)	28.7 (4.98)	0.97	29.7 (5.23)	30.4 (5.28)	0.24
Poverty Income Level	2.70 (1.82)	2.54 (1.79)	0.39	2.69 (1.72)	3.14 (2.36)	**0.04**
Race (%)						
White	81.8	85.1	0.31	85.4	85.7	0.38
Black	4.1	5.2	0.31	4.9	3.5	0.38
Hispanic Ethnicity	9.1	4.6	0.31	4.1	6.1	0.38
Other	4.9	5.2	0.31	5.7	4.8	0.38
Education (%)						
High school graduate or less	19.8	21.1	0.59	12.3	14.3	0.27
College graduate or more	80.2	78.9	0.59	87.7	85.7	0.27
Marital Status (%)						
Married	82.8	78.9	0.55	84.0	87.7	0.54
Widowed/Divorced/Separated	2.6	3.1	0.55	4.2	3.1	0.54
Never Married	14.7	18.0	0.55	11.8	9.1	0.54
Nulliparous (%)	27.5	25.5	0.66	29.8	31.3	0.76
Works for someone else full time^*^ (%)	33.3	39.2	0.27	43.5	30.0	**0.047**
Mother enrollment in WIC 2-mo (%)	28.7	26.4	0.65	28.2	20.7	0.13
Servings of Fruit^a^ (Mean (SE))	2.47 (0.17)	1.77 (0.20)	**0.0006**	1.70 (0.17)	2.29 (0.12)	**0.002**
Servings of Vegetables	2.76 (0.10)^b^	1.25 (0.06)^a^	**< 0.0001**	2.70 (0.10)^b^	4.25 (0.07)^c^	**< 0.0001**
Servings of Dairy	2.99 (0.11)^ab^	3.21 (0.06)^a^	0.08	2.93 (0.11)^ab^	2.71 (0.08)^b^	0.07
Servings of whole grains^a^	3.03 (0.06)	2.96 (0.03)	0.28	2.92 (0.05)	3.09 (0.04)	**0.01**
Grams of sugar^a^	49.8 (0.23)^b^	50.8 (0.13)^a^	**< 0.0001**	50.0 (0.23)^b^	48.8 (0.17)^c^	**< 0.0001**
Grams of discretionary fat^a^	20.3 (1.13)	21.3 (0.65)	0.42	22.9 (1.12)	23.6 (0.81)	0.64

^a^ This is an average of the pre-post

^b^ This variable is defined as 0 (fulltime homemaker, disabled student, etc. and not employed, retired and not employed, temporarily unemployed), 1 = self-employed, works for someone else part time only, or 2 = works for someone else full time)

Boldface indicates statistical significance (*p* < 0.05)

*WIC* Women Infant and Children

Poverty Income Level: Indicates federal poverty level

^c^ Dietary variables were not considered possible control variables

Women who *improved* their vegetable intake between the pre-and postnatal periods, compared to the *stable inadequate* group, smoked fewer cigarettes during pregnancy (0.48 ± 2.2 vs. 1.1 ± 3.9; *p* = 0.02) (Table 2). Women in the *reduced* vegetable group intake, compared to the *stable adequate* group, women had lower pregnancy weight gain (32.4 ± 14.8 vs. 26.7 ± 14.7 lbs.; *p* = 0.0007).

**Table 2:** Maternal Characteristics by Energy-Adjusted Change in Vegetable Intake in the IFPS II

Mean (SD) ** Residual adjusted + 2.5						
	Improved Vegetable Intake (*n* = 121)	Stable Inadequate (*n* = 370)	*p*-value	Reduced Vegetable Intake (*n* = 123)	Stable Adequate (*n* = 233)	*p*-value
Pre-pregnancy BMI	26.0 (5.60)	25.8 (6.33)	0.82	27.3 (6.86)	26.6 (6.61)	0.36
Pregnancy weight gain (lb)	30.9 (12.3)	30.2 (12.48)	0.62	26.7 (14.70)	32.4 (14.8)	**0.0007**
Cigarettes smoked	0.48 (2.20)	1.14 (3.89)	**0.02**	0.82 (2.91)	0.40 (2.13)	0.16
Breastfeeding Duration (months)	5.9 (4.8)	5.6 (4.5)	0.62	6.2 (5.1)	6.80 (4.8)	0.28
Paid and unpaid maternity leave (wks.)	8.95 (13.2)	9.41 (8.58)	0.80	10.1 (7.09)	8.70 (8.18)	0.28
GDM in pregnancy (%)	4.59	6.49	0.47	11.1	7.6	0.29
PPD Score	8.08 (3.15)	8.41 (3.22)	0.35	8.80 (3.27)	8.37 (3.39)	0.27
Received Information about diet from health professional	83.1	80.9	0.61	82.8	84.1	0.76
Received Information about diet from WIC	30.8	30.2	0.91	28.9	28.3	0.91

Boldface indicates statistical significance (*p* < 0.05)

*GDM* Gestational Diabetes Mellitus, *PPD* Postpartum Depression

Separate multivariable logistic regression models were used to examine sociodemographic predictors of either *improved* (versus *stable inadequate*) and *reduced* vegetable intake (versus *stable adequate*). Final models included only significant predictors. No sociodemographic factors were significantly associated with *improved* vegetable intake. However, greater maternal employment significantly increased the odds of *reduced* vegetable intake from pregnancy to the postpartum period compared to women who were not employed (OR = 1.64; 95%CI 1.14–2.36) in fully adjusted models controlling for age, PIR, smoking, weight gain during pregnancy and paid maternity leave (Table 3).

**Table 3:** Sociodemographic Predictors of Reduced and Improved Vegetable Intake from the Pre- to Post-natal period

	Odds Ratio (95% CI)	
	Reduced Vegetable Intake vs. Stable Adequate	Improved Vegetable Intake vs. Stable Inadequate
Employment^a^	1.31 (1.01–1.70)	0.92 (0.72–1.17)
Model 1		
Employment^a^	1.33 (1.02, 1.74)	0.90 (0.70–1.15)
Age	0.97 (0.93–1.02)	1.02 (0.98–1.07)
Model 2		
Employment^a^	1.51 (1.14, 2.02)	0.87 (0.67–1.14)
Age	0.98 (0.94–1.03)	1.02 (0.97–1.06)
Poverty Income Ratio	0.84 (0.73–0.97)	1.05 (0.92–1.20)
Model 3		
Employment^a^	1.52 (1.13–2.04)	0.85 (0.65–1.11)
Age	0.98 (0.93–1.03)	1.02 (0.97–1.07)
PIR	0.87 (0.75–1.00)	1.07 (0.93–1.23)
Weight gain in pregnancy	0.97 (0.96–0.99)	1.01 (0.99–1.03)
Final Model		
Employment^a^	1.64 (1.14, 2.36)	0.88(0.64–1.22)
Age	0.98 (0.93, 1.03)	1.03(0.98–1.08)
Smoking	1.02 (0.93,1.13)	0.96 (0.89–1.04)
PIR	0.88 (0.76, 1.03)	1.01 (0.86–1.17)
Weight gain in pregnancy	0.97 (0.96, 0.99)	1.01 (0.99–1.03)
Weeks of paid maternity leave	0.96 (0.89, 1.03)	0.98 (0.92–1.05)

^a^ This variable is defined as 0 (fulltime homemaker, disabled student, etc. and not employed, retired and not employed, temporarily unemployed), 1 = self-employed, works for someone else part time only, or 2 = works for someone else full time)

*PIR* Poverty Income Ratio

**Fig. 1:**
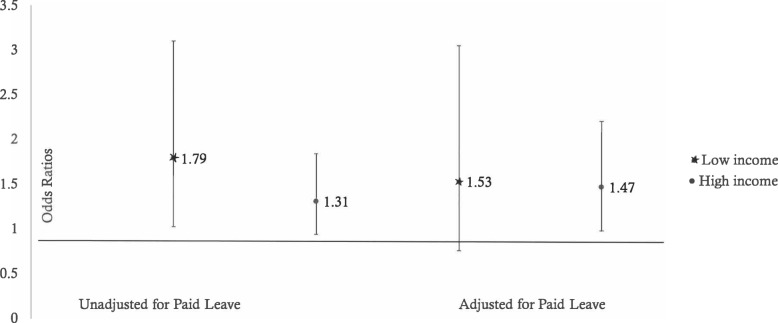
Fulltime Employment and Odds of Reduced Vegetable Intake Stratified by Poverty Income ratio and Unadjusted and Adjusted for Paid Leave

The strength of association between employment and r*educed* vegetable intake varied among women of higher socioeconomic status (PIR > 1.85) versus lower socioeconomic status (PIR < 1.85). For low income women, an increase in employment status was associated 1.79 times greater odds of *reduced* vegetable intake from pregnancy to the postpartum period after adjusting for age and gestational weight gain (OR = 1.79; 95%CI 1.03–3.1). For those that had higher incomes, there was no significant association between increasing employment status and the odds of *reduced* vegetable intake (OR = 1.31; 95%CI 0.94–1.84). However, after adjusting for weeks of paid maternity leave, the association between increasing employment and the odds of *reduced* vegetable intake was similar between higher and lower income women and no longer statistically significant among lower income women (OR: 1.53; 95%CI: 0.76–3.05) (Fig. 1).

We also examined dietary predictors of *improved* and *reduced* vegetable intake. With respect to *improved* vegetable intake, in analyses adjusted for age and other dietary components, a 1-unit increase in mean fruit intake was associated with a 12% increased odds of *improved* vegetable intake (OR = 1.12; 95%CI:1.00–1.21). A 1-unit increase in mean sugar (OR = 0.82; 95%CI: 0.73–0.91) or mean dairy (OR = 0.82; 95%CI: 0.73–0.91) was associated with a nearly 20% reduced odds of *improved* vegetable intake. A 1-unit increase in mean fruit intake was associated with a 15% reduced odds of *reduced* vegetable intake (*p* < 0.05). Conversely, a 1-unit increase in sugar (OR = 1.31; 95%CI: 1.17–1.48) or dairy (OR = 1.30; 95%CI: 1.03–1.63) was associated with a 30% increased odds of *reduced* vegetable intake (Table 4).

**Table 4:** Maternal and Dietary Predictors of Reduced and Improved Maternal Vegetable Intake from the Pre- to Post-natal period

	Odds Ratio (95% CI)	
	Reduced Vegetable Intake vs. Stable Adequate	Improved Vegetable Intake vs. Stable Inadequate
Mean fruit intake	0.81 (0.71–0.94)	1.18 (1.07, 1.31)
Model 1		
Mean fruit intake	0.81 (0.71–0.94)	1.18 (1.07–1.31)
Age	0.97 (0.93–1.02)	1.00 (0.96–1.05)
Model 2		
Mean fruit intake	0.83 (0.72–0.97)	1.14 (1.03–1.27)
Age	0.98 (0.94–1.03)	1.00 (0.95–1.04)
Mean sugar	1.29 (1.14–1.45)	0.84 (0.76–0.93)
Final Model		
Mean fruit intake	0.85 (0.73–0.98)	1.12 (1.00, 1.24)
Age	0.98 (0.94–1.03)	0.99 (0.95–1.04)
Mean sugar	1.31 (1.17–1.48)	0.82 (0.73–0.91)
Mean dairy	1.30 (1.03–1.63)	0.81 (0.67–0.98)

## Discussion

The goal of this study was to 1) explore the changes in vegetable intake between pregnancy and the postnatal period in a large, prospective cohort and 2) explore maternal and sociodemographic factors that are associated with these changes. Within this national sample of pregnant women, we found that 44% of women were not meeting vegetable recommendations during pregnancy or the postpartum period, 28% met recommendations at both time points, and the remaining 28% experienced changes. Notably, 15% of women who consumed the recommended servings of vegetables during pregnancy transitioned to not meeting the recommendation at 4-months postpartum. Importantly, changes in vegetable intake appear to align with changes in other important food groups such as fruits and added sugars; mean fruit intake was associated increased odds of *improved* vegetable intake while mean sugar intake was associated with decreased odds of *improved* vegetable intake. We also found that employed women were more likely to have *reduced* vegetable intake and that this association differed among high- and low-income women; for low-income women, employment was associated with greater odds of *reduced* vegetable intake, while there was no significant association among higher income women. However, the association between employment and vegetable intake was similar between higher and lower income women and no longer statistically significant among lower income women after adjusting for paid maternity leave.

Many of our results are consistent with existing literature. For example, overall, we found that almost half of women in this cohort are not meeting vegetable recommendations during and after pregnancy. This is similar to other studies whereby overall diet quality among both pregnant and non-pregnant women is suboptimal, with high intakes of sugary drinks, and low intakes of nutrient dense foods such as vegetables [35, 36]. Although previous studies in pregnant and postpartum women are limited in number, one showed that maternal macronutrient intake did not change drastically during this time period, which differs from our food-based analysis, where 28% of women improved or reduced their vegetable intake from the pregnancy to postpartum [20]. Findings from a qualitative study suggest that mothers believe they change their diets and eating habits after the birth of their child and that they turn towards unhealthier dietary habits [38]. The primary reason for changing their diet was feeling responsible for their baby’s digestive discomfort via their breastmilk, which narrowed their range of foods, time scarcity for food preparation and eating and disruptions in their usual eating routines. We found that the odds of *reduced* vegetable intake was greater with increasing intake of added sugar and dairy, supporting the hypothesis that energy-dense convenience foods may substitute for more nutrient-dense choices. Our findings suggest that there is a window of opportunity to help women maintain or improve their diet quality as they transition to becoming a parent.

The complex association we describe between maternal employment, socioeconomic status, paid leave, and diet quality warrants further exploration. Increasing maternal employment was associated with a 60% greater odds of *reduced* vegetable intake. Our findings are consistent with one study whereby mothers felt that going back to work reduced their diet quality [38]. This study, however, did not explore differences by income, and was conducted among Danish mothers who experience longer periods of maternity leave. We found that there was no association between employment status and *reduced* vegetable intake among higher income women whereas among lower income women increasing employment was associated with a greater odds of *reduced* vegetable intake from pregnancy to the postpartum period. However, after further adjustment for weeks of paid maternity leave, this association was similar between higher and lower income women and no longer statistically significant among lower income women suggesting that differential access to this resource may contribute to socioeconomic disparities in vegetable intake among pregnant women.

Maternal employment may be related to *reduced* vegetable intake among lower income women because of additional challenges associated with significant financial barriers. Employed mothers perceive that they have less time to prepare and procure meals and that changing demands on their time promotes a shift towards consuming more commercially prepared and fast foods [39]. Mothers also report psychological effects of employment such as higher levels of stress and depression, which may adversely influence diet quality [40]. This may be exacerbated among lower income women who both work for lower wages and manage other stressors associated with balancing financial strain and parenthood [41]. There may be differential access to nutrient-dense and affordable convenience foods for low-income women. Furthermore, low-income women may disproportionately participate in occupations that provide limited autonomy and power to employees, disempowering them to continue prioritizing more healthful dietary choices upon return to work [42]. In other words, the food-coping strategies available to lower income, fully employed women may differ from their higher income counterparts [41].

Although it is well established that food availability, access, cost, cultural preferences, and social roles are all important determinants of food choice [43], little research has explored modifiable factors that could favorably influence food choice without changing these underlying determinants. Our research adds to this by considering the impact of temporary support systems, like paid maternity leave, to support healthful food choices. Our finding that paid maternity leave appears to eliminate the disparity in vegetable intake between employed higher and lower income pregnant women after birth, highlights the importance of this temporary support. One study similarly found that the dietary intake of working women with children did not appear to be influenced by hours of employment [44], suggesting that perhaps it is not employment per se that is associated with reduced diet quality during the transition to motherhood but rather ensuring that that they have enough support to return to work.

Our study has some limitations that should be discussed. First, the IFPS II data was collected in 2007 and was among predominately White, educated, and non-diverse, limiting the generalizability to other race/ethnicities where rates of obesity are higher, and likely underestimating the strength of association observed. Diet was self-reported, which is subject to under or over reporting; however, the DHQ is considered a reasonably valid and reliable tool for this population. Additionally, height and weight were self-reported, which may have attenuated the associations between maternal BMI, gestational weight gain, and changes in vegetable intake. Finally, women were drawn from a self-selected consumer panel and not a random sample; hence, they may not be representative of the US population, although the sample was well distributed throughout the United States.

Several strengths of our study include the large national sample, two diet collection periods, and detailed information on sociodemographic and maternal characteristics. Additionally, examining changes in vegetable intake between pregnancy and postpartum provides unique insight into a critical window when a woman’s diet may change, which is important for the development of nutrition policy. While pregnant women may feel motivated and supported to make healthful food choices during pregnancy [37], our results suggest that additional support systems may be needed in the period immediately postpartum – especially among lower income women.

## Conclusion

Establishing a healthy diet as a woman transitions to becoming a mother is critical not only for her own health but for that of her child. While our results suggest that many pregnant and post-partum women are not consuming the recommended servings of vegetables each day, we also highlight a group of women for whom additional support could help them maintain adequate prenatal vegetable intake into the postpartum period. Systemic barriers including socioeconomic disadvantage are difficult to structurally change, and our results provide preliminary evidence for short-term support that may help reduce health disparities.

## Data Availability

Although the datasets used and/or analyzed during the current study are publicly available, an email should be sent to the following email address to request access: ifps@cdc.gov.

## Abbreviations

DHQ: Diet history questionnaire
IFPSII: Infant Feeding Practice Study II cohort
OR: Odds ratio
PIR: Poverty income ratio
WIC: Women, Infants and Children Program
